# Addressing Disturbance in Bodily Experience After Ventricular Assist Device Implantation: A Multicenter Randomized Controlled Trial of Curricular Psychological Support

**DOI:** 10.1111/aor.14996

**Published:** 2025-03-17

**Authors:** Wolfgang Albert, Hannah Spielmann, Sandra Semmig‐Koenze, Christoph Knosalla, Johanna Mulzer, Katharina Tigges‐Limmer, Christiane Kugler, Fabian Richter

**Affiliations:** ^1^ Deutsches Herzzentrum der Charité Department of Cardiothoracic and Vascular Surgery Berlin Germany; ^2^ Charité—Universitätsmedizin Berlin, Corporate Member of Freie Universität Berlin and Humboldt‐Universität Zu Berlin Berlin Germany; ^3^ DZHK (German Centre for Cardiovascular Research), Partner Site Berlin Berlin Germany; ^4^ Faculty of Medicine, Institute of Nursing Science University of Freiburg Freiburg Germany; ^5^ Leipzig Heart Center Leipzig Germany; ^6^ Heart and Diabetes Center North‐Rhine Westphalia, University Hospital of the Ruhr University Bochum Bad Oeynhausen Germany

**Keywords:** body image, disturbance in bodily experience, LVAD, psychological intervention, RCT, VAD, ventricular assist device

## Abstract

**Background:**

Disturbance in bodily experience (BE) after ventricular assist device (VAD) implantation is common. This study aims to investigate the effect of focused psychological support to improve BE in VAD patients.

**Methods:**

This national, multicenter, longitudinal study enrolled 140 VAD patients from four centers across Germany in a prospective, randomized controlled trial. Patients were randomized (1:1) to receive curricular and focused psychological intervention in the post‐implantation step‐down units after implantation (*n* = 70) or treatment as usual (*n* = 70). BE was assessed using the Bodily Experience Scale in VAD Patients (BE‐S) after implantation (baseline) and followed up 12 months later. Data were analyzed using mixed‐effects models.

**Results:**

VAD patients with disturbance in BE (BE‐S ≥ 2) after implantation (*n* = 43, 63.24%) benefit from the targeted intervention. Compared to the subsample of the control group (CG) patients with initial BE disturbance (*n* = 46, 69.7%), the intervention group (IG) displays a significantly stronger decrease from baseline to the 1‐year follow‐up (*p* = 0.01). Generally, women (*p* = 0.4) and emergently implanted patients (*p* = 0.24) show a smaller, albeit not significant, decrease in BE disturbance. All patients have high overall gratitude scores, which increase slightly but not significantly over time.

**Conclusion:**

The efficacy of targeted psychological support in reducing disturbance in BE among VAD patients is highlighted. Given the strong correlation between BE and other mental health domains, such as anxiety and depression, it is essential to address disturbances in BE to improve the overall mental health of VAD patients.

## Graphical abstract

Improving mental health in VAD patients: Focused psychological support mitigates disturbance in bodily experience after VAD implantation. Findings from a randomized controlled trial.

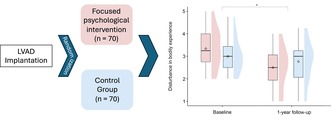

## 1 Background

The psychological impact of ventricular assist device (VAD) implantation is frequently neglected yet constitutes a crucial factor. Reduced mental health affects the quality of life and contributes to poor adherence, frequent comorbidities, and ultimately worsens the long‐term outcome of the treatment. However, there is a paucity of studies investigating psychological variables in the post‐VAD period, let alone longitudinal studies.

In a recent work, we investigated the bodily experience (BE) in VAD patients for the first time and obtained revealing insights into the prevalence of disturbance in BE. Further, we identified female and emergently implanted patients as particularly vulnerable cohorts [1]. Building on this, we now aim to delve further into the psychological processes associated with BE after VAD implantation by analyzing their dynamics over time. Findings in this area carry important implications for the appropriate psychological aftercare of VAD patients.

When perceiving our own bodies, we are both subject and object of our perception at the same time. This epistemological intertwining makes the experience and representation of our physical self a topic that has long been of ongoing great interest. Some suggested, as the vital organs are constantly monitored for homeostatic regulation, that the continuous processing of interoceptive signals lays the foundation for bodily self‐consciousness and bodily experience [2, 3, 4]. Another line of research manipulates a crucial feature of bodily experience, namely body ownership, through body illusions which create multisensory conflicts, such as the Rubber‐Hand Illusion [5] or the body‐swapping experience [6, 7]. Further, clinicians focus on disorders of bodily experiences in neurological or psychiatric contexts, such as depersonalization [8], anosognosia [9], phantom limb [10], or eating disorders [11]. We define BE as a broad and general concept that covers all cognitive and affective processes related to the subjective experience of one's own body [12, 13, 14]. This involves the processing and integration of exteroceptive, interoceptive, proprioceptive, and vestibular afferent input, that is, the entirety of sensory perception. Furthermore, during the integration of this information and in order to form a comprehensive and ongoing bodily experience, implicit and explicit stored “top‐down” self‐representations exert their modifying effects through higher cognitive processes [15, 16]. Following this interference model, BE is conceptualized as an epiphenomenon that arises from the interplay of multisensory integration and cognition.^1^

This study employed a randomized controlled trial to investigate whether early and targeted psychological interventions to promote integration and adaptation of the device can lead to a lasting reduction of disturbance in BE. In the context of device implantation, *integration* refers to the psychological processes by which patients incorporate the VAD into their sense of self and accept the device as an integral aspect of their body identity. *Adaptation* pertains to the practical adjustments and behavioral modifications patients make to accommodate the presence of the VAD. It includes modifications in activities and routines, gaining independence from caregivers, and adjusting to a new state of normalcy [17, 18].

We hypothesized the intervention group (IG) to have a significantly greater decrease of BE disturbance after 1 year compared to a control group (CG) receiving usual care. Usual care in the CG means that patients received psychological support only upon request or in cases of specific need. Based on our previous results, which identified female and emergently implanted patients as risk groups [1], we further expected that women as well as patients with higher acuity (emergently implanted patients) would have more disturbance in BE and show less decrease over time. The course of the patients' gratitude was explored without prior assumptions.

## 2 Methods

### 2.1 Study Design

This non‐blinded block‐randomized controlled trial is part of a national interventional study (SELMA) [19]. Patients were simultaneously recruited at four participating university‐based heart centers across Germany. The baseline assessments prior to the psychological intervention were conducted after discharge from the intensive care unit, between January 2021 and October 2021. The follow‐up assessment was performed 1 year later. The stratified block randomization procedure, allocation ratio 1:1, was based on sex and center. For the CONSORT study flow diagram and checklist, see Supporting Information.

The study was conducted in compliance with the Helsinki Declaration and the European General Data Protection Regulation (EU‐GDPR). The complete study protocol obtained approval from the institutional ethics board of the coordinating center (EK‐Nr. 304/19) and was subsequently confirmed by the review boards of the participating centers. The study protocol has been registered on clinicaltrials.gov (NCT04526964).

### 2.2 Study Population

Patients who met the inclusion criteria (sufficient cognitive and linguistic abilities, stable postoperative condition indicated by transfer from the intensive care unit to the step‐down unit, legal age [≥ 18 years]) received detailed information about the study through authorized professionals at the step‐down units after VAD implantation. Upon written consent to participate following an appropriate reflection period, pseudonymized randomization was performed (see Supporting Information for a CONSORT flow diagram).

### 2.3 Intervention

The intervention focusing on bodily experience took place during the postimplant hospitalization for clinically stable participants being assigned to the IG. The intervention consisted of two sessions with trained psychologists. The contents of the sessions were manualized and focused on adapting to life with the VAD and integrating the VAD into the patients' body identity. This included cognitive‐behavioral and psychodynamic techniques, such as imagination, emotional acceptance, coping facilitation and resource activation. Specifically, during the 30–45 min sessions, various techniques were used, such as: giving the VAD a name to psychodynamically reduce the fear of the foreign object and promote emotional acceptance; strengthening the alliance between the VAD and the heart through interoceptive sensing; preventing a negative body image by enhancing body‐related self‐confidence, accepting the altered physical appearance, and providing tips on clothing with the VAD; fostering awareness of one's own (physical) strength; setting positive recovery goals; developing coping strategies for potential challenges related to living with the VAD; and raising awareness of social network resources. IG patients were provided with a refresher session at their first outpatient follow‐up visit at the respective center, approximately 3 months after implantation. Patients in the CG received treatment as usual, that is, they only received psychological support if requested or in cases of particular need. For a standardized delivery of treatment at all four sites, a manualized curriculum was developed, and all involved staff members participated beforehand in a 1‐day train‐the‐trainer workshop (for details see Kugler et al. [19]).

### 2.4 Measures

*Bodily experience and gratitude*. To survey patients' BE, the Bodily Experience Scale (BE‐S) was utilized [20]. The scale comprises four items specifically designed to assess disturbances in bodily experience among patients with a VAD. The scale ranges from 1 to 5, with higher values indicating more disturbances. An additional item (Gratitude) assesses patients' gratitude on a scale from 1 to 5, with 5 representing the highest levels of gratitude. This item is included due to its relevance. Additionally, it captures patients' attitudes toward the VAD on an abstract level, which can differ from more concrete symptom‐related judgments. See Table 1 for a description of the individual items.

**TABLE 1:** Bodily experience scale in VAD patients (BE‐S).

Item	Question	Dimension
1	I feel well in my own body with the VAD	Bodily experience
2	I have the feeling of being myself with the VAD	
3	The VAD feels strange to me	
4	I have integrated the VAD into my body	
Extra item	I am grateful for the VAD	Gratitude

The *demographic* data was collected by means of questionnaires; *somatic* data was taken from the patient records.

### 2.5 Statistical Analyses

A linear mixed‐effects model analysis was conducted to investigate the influence of the fixed effects *group* (intervention vs. control), *timepoint* (baseline vs. 1‐year follow‐up), *sex* (male vs. female) and *patient acuity* (elective vs. emergent) on the dependent variable *disturbance in BE*. Of particular interest here were the interactions of the various fixed effects with the factor *timepoint*. The model included a random intercept for each *patient* to account for the repeated measurements within individuals. *Age* and *center* were included as covariates.

We refrained from calculating three‐way interactions (e.g., timepoint × group × sex) due to the small number of participants remaining in some subgroups.

To pay particular attention to only those patients showing signs of disturbance in bodily experience after implantation, this mixed‐effects model analysis was repeated with the subgroup of patients who had baseline BE‐S values of ≥ 2. In addition, the whole‐group analysis was repeated with gratitude as the dependent variable instead of disturbance in BE.

Pearson correlation coefficients were calculated separately for both groups between the change score of the disturbance in BE and the ages of the patients. Point‐biserial correlations were calculated between the change score of the disturbance in BE and the driveline infections, also separately for both groups.

Data were considered incomplete and excluded from the respective analysis at the corresponding time point if one or more items of the BE‐S were not answered. We opted against listwise case deletion, as mixed‐effects models can handle randomly missing data at either time point using maximum likelihood estimations, which increases the statistical power and mitigates the risk of biased results [21]. Analyses were conducted in R [22], the mixed‐effects models were fitted using the lme4 package [23]. Figures were created with the package ggplot2 [24]. The significance level for two‐tailed testing was set at *p* < 0.05.

## 3 Results

### 3.1 Descriptive Results

**FIGURE 1:**
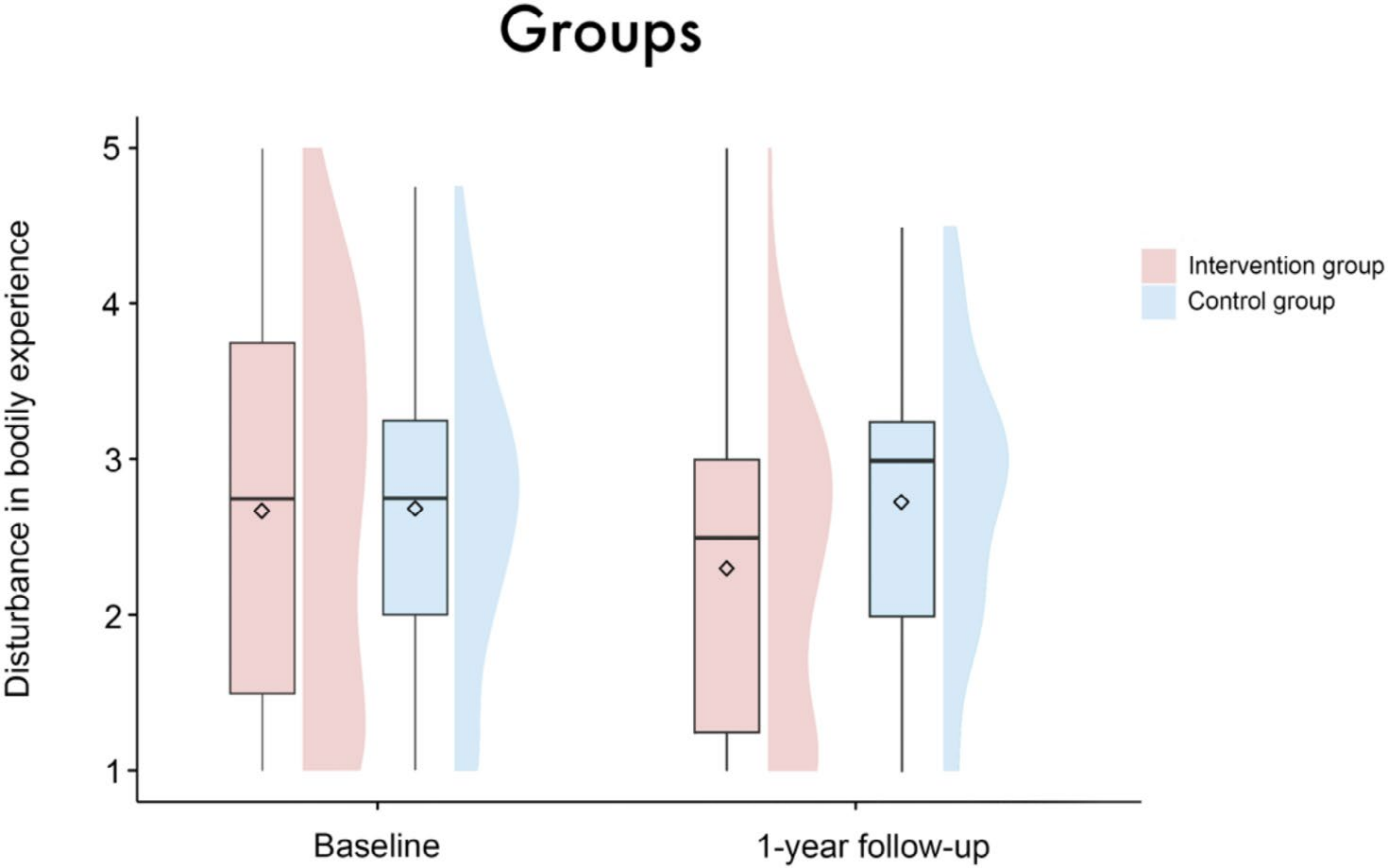
Boxplots with split‐half violin plots visualizing the disturbance in BE for both groups at both time points. The diamond represents the mean value. No significant main effect of group (*β* = 0.01 [95%‐CI: −0.35, 0.38], SE = 0.19, *t* = 0.07, *p* = 0.95) or the interaction between group × time point (*β* = 0.35 [95%‐CI: −0.08, 0.79], SE = 0.23, *t* = 1.55, *p* = 0.12) was found. [Color figure can be viewed at wileyonlinelibrary.com]

In the IG, there was a reduction in the mean disturbance in BE from 2.66 (SD = 1.23) at baseline to 2.31 (SD = 1.0) at the one‐year follow‐up. In contrast, the control group exhibited a slight increase in mean disturbance in BE from 2.67 (SD = 0.95) to 2.73 (SD = 0.88); see also Figure 1. The mean gratitude scores increased in both groups from baseline (intervention group: 4.41 (SD = 0.99), control group: 4.03 (SD = 1.2)) to the one‐year follow‐up (intervention group: 4.53 (SD = 0.93), control group: 4.2 (SD = 0.98)); see Figure 5. For data quality, demographics, and characteristics of the study sample, see Table 2.

**FIGURE 2:**
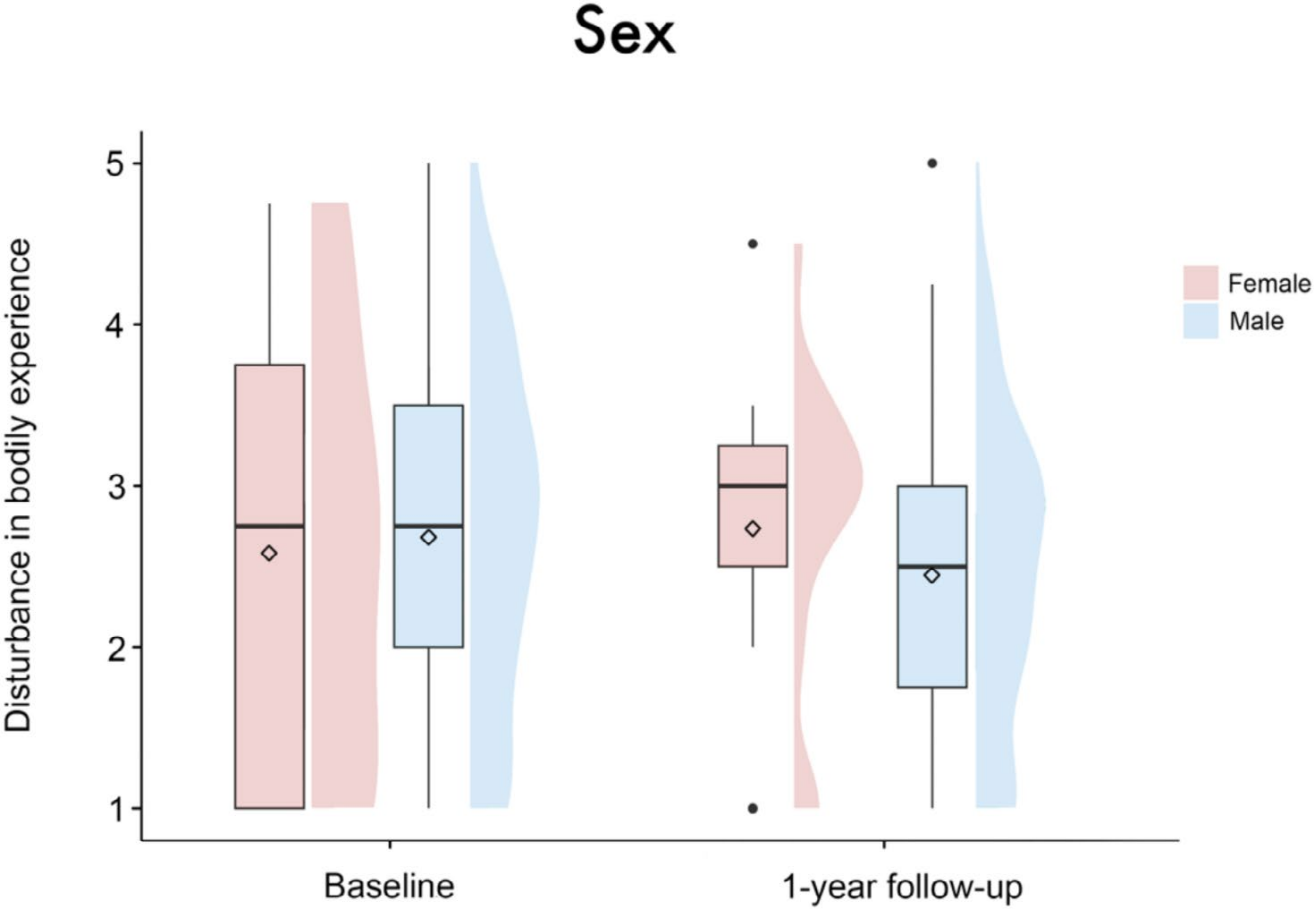
Boxplots with split‐half violin plots visualizing the disturbance in BE for male and female patients at both timepoints. The diamond represents the mean value. No significant main effect of sex (*β* = 0.1 [95%‐CI: −0.38, 0.57], SE = 0.25, *t* = 0.39, *p* = 0.7) or the interaction between sex and timepoint (*β* = −0.25 [95%‐CI: −0.84, 0.32], SE = 0.3, *t* = −0.85, *p* = 0.4) was found. [Color figure can be viewed at wileyonlinelibrary.com]

**TABLE 2:** Demographics.

Characteristics	Mean (SD) or *n* (%)		*p*
	Intervention group	Control group	
Drop‐Outs	2 (2.86%)	4 (5.71%)	
Gender			0.93
Male	55 (80.88%)	53 (80.3%)	
Female	13 (19.12%)	13 (19.7%)	
Patients with complete data at baseline	63 (92.65%)	53 (80.3%)	
Patients with complete data at 1 year follow‐up	56 (82.35%)	44 (66.67%)	
Age	57.84 (10.97)	57.83 (11.02)	0.99
Patient acuity			0.94
Elective	56 (82.35%)	54 (81.82%)	
Emergent	12 (17.65%)	12 (18.18%)	
Marital status			0.17
Single	17 (25%)	23 (35.94%)	
Married or partnered	51 (75%)	41 (64.06%)	
Living situation			0.17
Alone	12 (17.65%)	18 (27.69%)	
Not alone	56 (82.35%)	47 (72.31%)	
Education (highest)			0.96
Secondary school	24 (35.82%)	23 (35.94%)	
High school	4 (5.97%)	3 (4.69%)	
Vocational education	28 (41.79%)	29 (45.31%)	
University degree	11 (16.42%)	9 (14.06%)	
Kids			0.65
Yes	22 (32.35%)	19 (28.79%)	
No	46 (67.65%)	47 (71.21%)	

*Note:* Discrepancies to total are due to missing values.

Abbreviations: M, mean; *n*, number of patients; SD, standard deviation.

### 3.2 Results on BE

**FIGURE 3:**
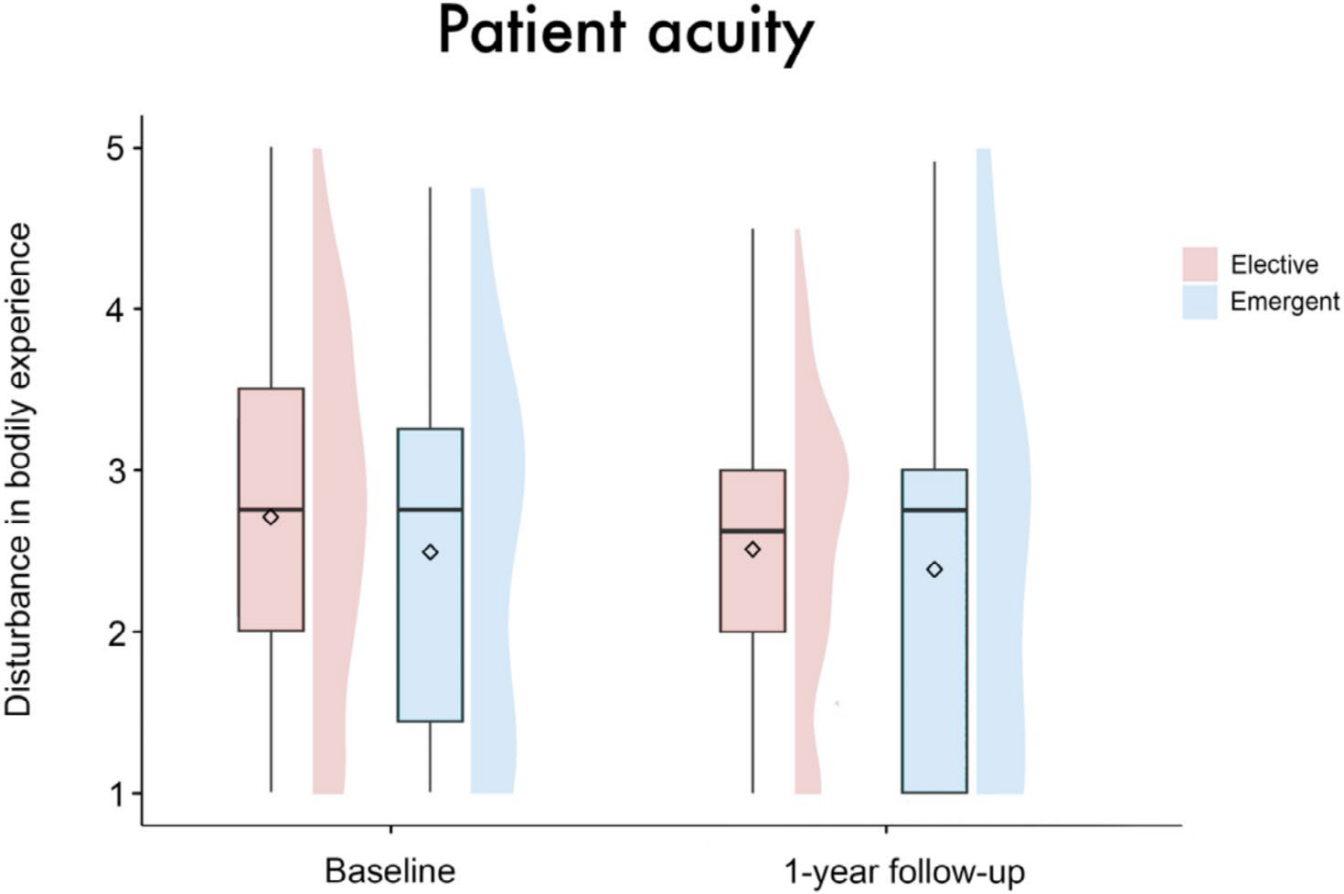
Boxplots with split‐half violin plots visualizing the disturbance in BE for elective and emergent patients at both time points. The diamond represents the mean value. No significant main effect of patient acuity (*β* = −0.32 [95%‐CI: −0.81, 0.18], SE = 0.26, *t* = −1.23, *p* = 0.22) or the interaction between patient acuity × time point (*β* = 0.36 [95%‐CI: −0.25, 0.96], SE = 0.31, *t* = 1.17, *p* = 0.24) was found. [Color figure can be viewed at wileyonlinelibrary.com]

The linear mixed‐effects model analysis revealed no significant main effect of group (*β* = 0.01 [95%‐CI: −0.35, 0.38], SE = 0.19, *t* = 0.07, *p* = 0.95), timepoint (*β* = −0.62 [95%‐CI: −2.1, 0.87], SE = 0.77, *t* = −0.82, *p* = 0.42), sex (*β* = 0.1 [95%‐CI: −0.38, 0.57], SE = 0.25, *t* = 0.39, *p* = 0.7) or patient acuity (*β* = −0.32 [95%‐CI: −0.81, 0.18], SE = 0.26, *t* = −1.23, *p* = 0.22) on disturbance in BE. There were also no significant results regarding the interactions of these variables with the factor timepoint (group × timepoint: *β* = 0.35 [95%‐CI: −0.08, 0.79], SE = 0.23, *t* = 1.55, *p* = 0.12; sex × timepoint: *β* = −0.25 [95%‐CI: −0.84, 0.32], SE = 0.3, *t* = −0.85, *p* = 0.4; patient acuity × timepoint: *β* = 0.36 [95%‐CI: −0.25, 0.96], SE = 0.31, *t* = 1.17, *p* = 0.24), see Figures 1, 2, 3. Age (*β* = −0.02 [95%‐CI: −0.03, −0.001], SE = 0.01, *t* = −2.14, *p* = 0.03), but not center (*β* = −0.07 [95%‐CI: −0.2, 0.06], SE = 0.07, *t* = −1.0, *p* = 0.32) emerged as a significant covariate in the model.

**FIGURE 4:**
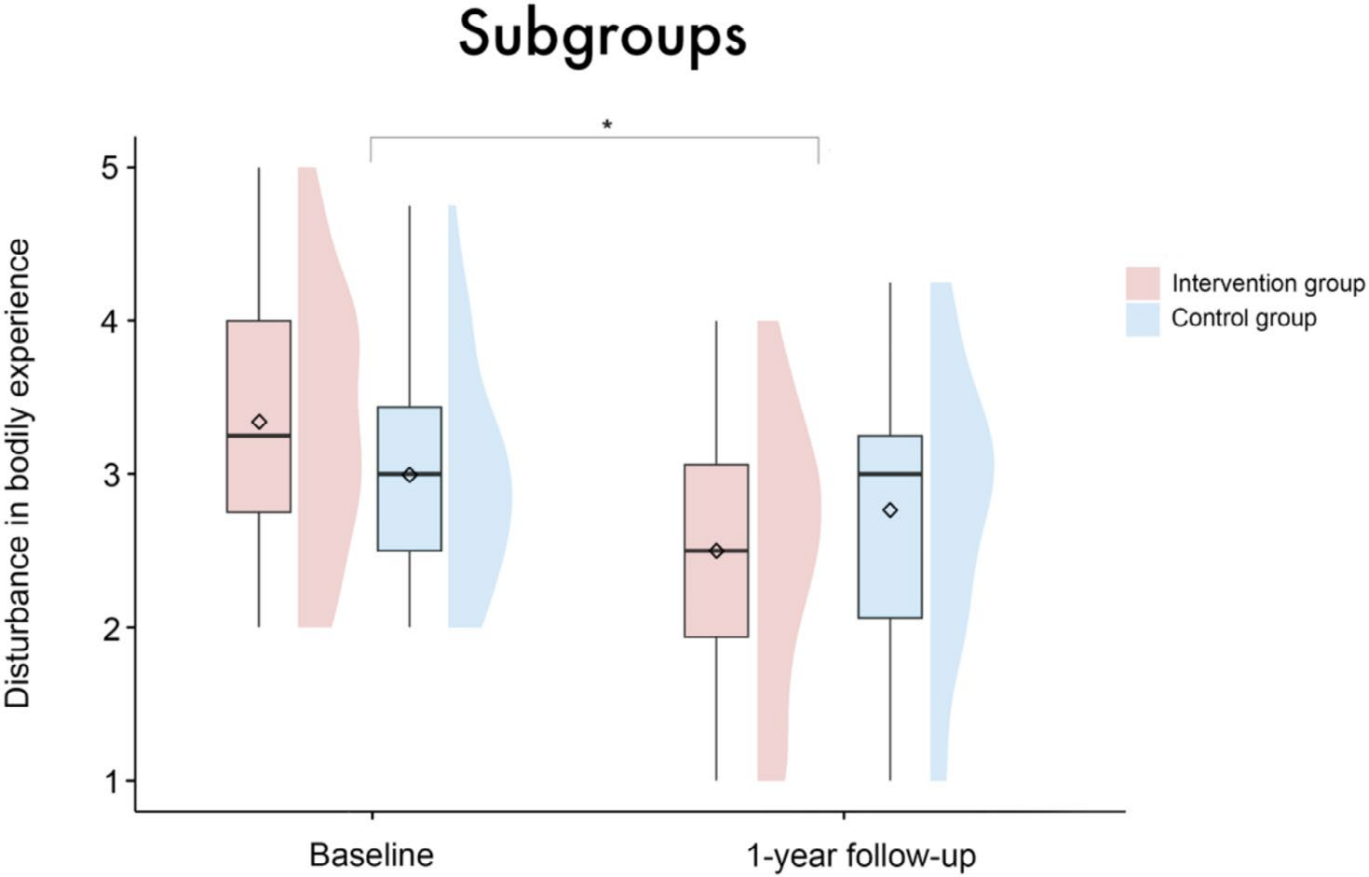
Boxplots with split‐half violin plots visualizing the disturbance in BE for both groups at both timepoints. Only the subgroup of patients with baseline BE‐S values of ≥ 2 was considered. The diamond represents the mean value. No significant main effect of group (group: *β* = −0.33 [95%‐CI: −0.67, 0.0004], SE = 0.18, *t* = −1.9, *p* = 0.06) but a significant interaction between group × timepoint (*β* = 0.59 [95%‐CI: 0.15, 1.05], SE = 0.23, *t* = 2.56, *p* = 0.01) was found. [Color figure can be viewed at wileyonlinelibrary.com]

The picture changed if only the subgroup of patients with disturbance in BE at baseline is considered (IG *n* = 43, CG *n* = 46). Note that there was no significant age or sex difference between the subgroups; for a complete overview of the characteristics of these subgroups, see Table S1. Here, a significant interaction between group and timepoint was observed (*β* = 0.59 [95%‐CI: 0.15, 1.05], SE = 0.23, *t* = 2.56, *p* = 0.01), expressing a significantly stronger decrease in the IG compared to the CG from baseline to the 1‐year follow‐up (see Figure 4). The main effects for itself (group: *β* = −0.33 [95%‐CI: −0.67, 0.0004], SE = 0.18, *t* = −1.9, *p* = 0.06; timepoint: *β* = −0.97 [95%‐CI: −2.59, 0.68], SE = 0.85, *t* = −1.14, *p* = 0.26; sex: *β* = −0.15 [95%‐CI: −0.61, 0.31], SE = 0.24, *t* = −0.62, *p* = 0.54; patient acuity: *β* = 0.11 [95%‐CI: −0.39, 0.62], SE = 0.27, *t* = 0.43, *p* = 0.67) and the other interactions (sex × timepoint: *β* = −0.33 [95%‐CI: −0.97, 0.30], SE = 0.33, *t* = −0.99, *p* = 0.33; patient acuity × timepoint: *β* = 0.16 [95%‐CI: −0.59, 0.88], SE = 0.38, *t* = 0.41, *p* = 0.68) were not significant. None of the covariates had a significant effect (age: *β* = −0.01 [95%‐CI: −0.02, 0.002], SE = 0.01, *t* = −1.66, *p* = 0.1; center: *β* = −0.01 [95%‐CI: −0.13, 0.11], SE = 0.06, *t* = −0.15, *p* = 0.88).

**FIGURE 5:**
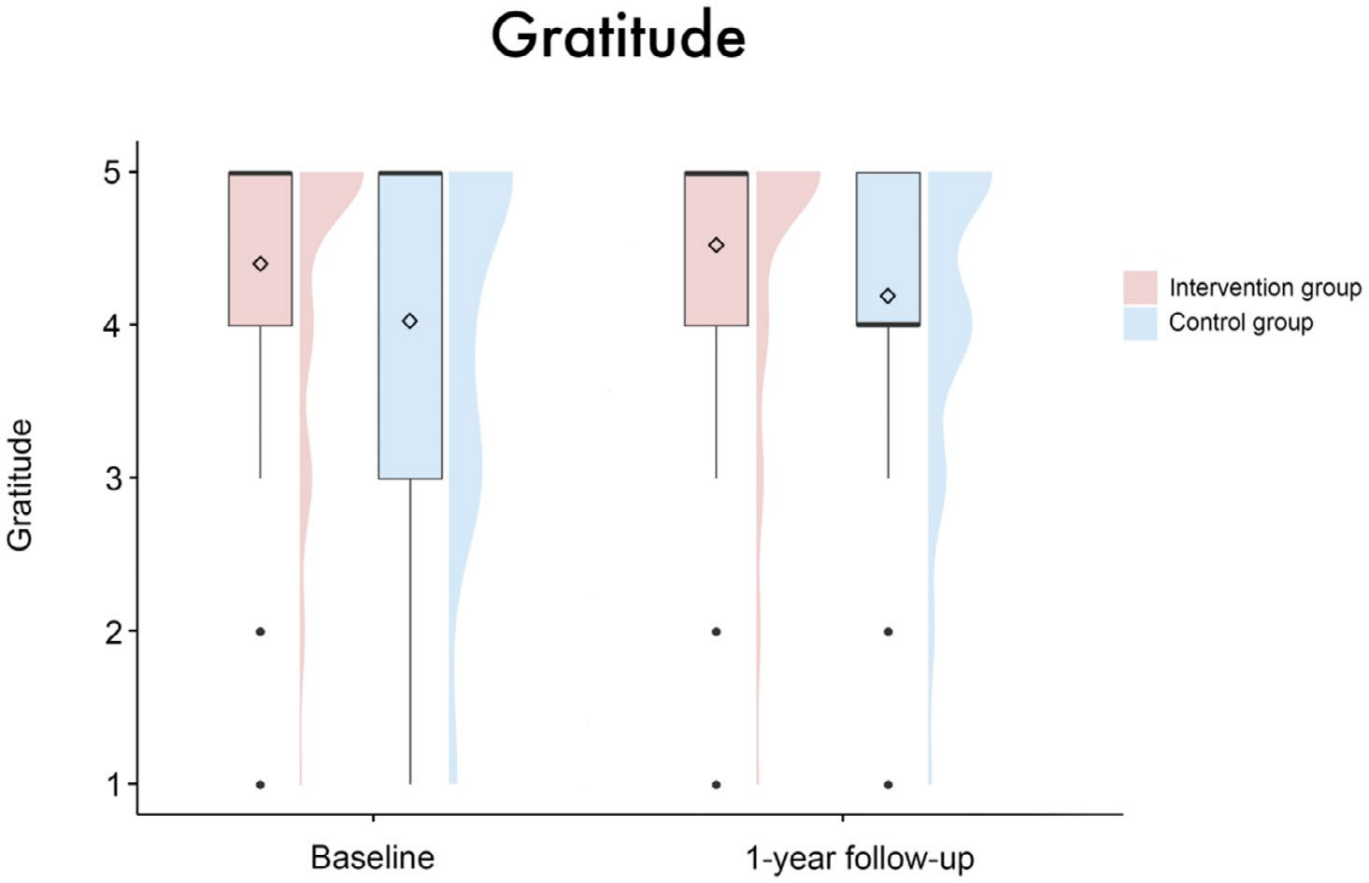
Boxplots with split‐half violin plots visualizing the gratitude scores for both groups at both timepoints. The diamond represents the mean value. A significant main effect of group (*β* = −0.37 [95%‐CI: −0.73, −0.01], SE = 0.19, *t* = −1.99, *p* = 0.048) but no significant effect for the interaction between group*timepoint (β = 0.04 [95%‐CI: −0.45, 0.53], SE = 0.25, *t* = 0.16, *p* = 0.87) was found. [Color figure can be viewed at wileyonlinelibrary.com]

### 3.3 Results on Gratitude

The linear mixed‐effects model analysis regarding gratitude disclosed a significant main effect of group (*β* = −0.37 [95%‐CI: −0.73, −0.01], SE = 0.19, *t* = −1.99, *p* = 0.048), as the scores for gratitude in the IG were higher than those of the CG at both time points. No significant effects were found for time point (*β* = −0.1 [95%‐CI: −1.76, 1.58], SE = 0.86, *t* = −0.12, *p* = 0.91), sex (*β* = −0.21 [95%‐CI: −0.67, 0.26], SE = 0.24, *t* = −0.85, *p* = 0.39) or patient acuity (*β* = 0.03 [95%‐CI: −0.46, 0.52], SE = 0.26, *t* = 0.12, *p* = 0.9). There were no interaction effects (group × timepoint: *β* = 0.04 [95%‐CI: −0.45, 0.53], SE = 0.25, *t* = 0.16, *p* = 0.87; sex × timepoint: *β* = 0.17 [95%‐CI: −0.47, 0.81], SE = 0.33, *t* = 0.51, *p* = 0.61; patient acuity × timepoint: *β* = −0.09 [95%‐CI: −0.78, 0.59], SE = 0.35, *t* = −0.24, *p* = 0.81). None of the covariates had a significant effect (age: *β* = −0.01 [95%‐CI: −0.02, 0.01], SE = 0.01, *t* = −0.42, *p* = 0.68; center: *β* = −0.03 [95%‐CI: −0.16, 0.09], SE = 0.06, *t* = −0.49, *p* = 0.63).

### 3.4 Correlation Analyses

The correlation between patient age and the change in BE‐S score from baseline to the 1‐year follow‐up in the IG was *r* (47) = 0.02, *p* = 0.89. In the CG, it was *r* (34) = −0.06, *p* = 0.72. Similarly, the point‐biserial correlations between the occurrence of a driveline infection during the first year and the BE‐S score were not significant in either group (IG: *r* (47) = −0.02, *p* = 0.91; CG: *r* (34) = 0.12, *p* = 0.47).

## 4 Discussion

We investigated the effect of focused psychological support after VAD implantation. Our results did not support the main hypothesis, namely that the focused psychological intervention would have a significant, positive effect on disturbance in bodily experience. However, the results do indicate that patients who show signs of disturbance in BE after implantation benefit from the focused intervention. Compared to the CG, these patients have significantly less disturbance in BE 1 year after implantation. This subgroup analysis is comprehensible and necessary, as patients without initial disturbances in body experience have no room for further improvement. In line with the existing literature, this subgroup finding again points to a disturbance in BE after VAD implantation [1, 25, 26, 27], but also offers targeted therapeutic support as an effective clinical tool for this patient cohort. While a profound impact of VAD implantation on patients' psychological and social functioning has been identified in the past [1, 17, 25, 26, 27, 28, 29, 30], this is the first study to systematically evaluate the effect of a standardized psychological intervention on BE in VAD patients. The intervention appears to particularly support the process of adaptation and gradual accommodation to the device [4, 18].

The VAD is a life‐sustaining device residing both inside and outside the patients' body. With both its intracorporal as well as peripheral parts, it represents equally an invisible, foreign as well as a visible, familiar object. Building on this, we propose a theoretical distinction between two parallel processes that we addressed during the intervention, namely *integration* and *adaptation*. Adaptation refers to the visible components outside of the body, such as the driveline, the controller unit and the battery packs. These parts affect the patients' daily functioning and autonomy, for example, attention to continuous power supply [17], personal hygiene [31], and sexuality [32, 33]. Here, patients need to adapt their behavior and lifestyle to accommodate the presence of these components. This includes modifications in activities, daily routines, and social interactions to manage the practical aspects of living with the VAD, such as diligent wound care [17, 18].

Integration, on the other hand, relates to the components of the VAD inside the patients' body. With the assist implantation, the patients' physical and personal integrity becomes dependent on a mechanical device. Therefore, device integration aims at the psychodynamic processes by which patients incorporate the VAD into their sense of self. It encompasses the acceptance and incorporation of the device as an important aspect of their identity and existence. Here, reference can be drawn to the integration of donor organs [34, 35].

This study demonstrates that patients with an initial disturbance in BE benefit from psychological support to promote adaptation to and integration of the device. It therefore consequently builds on a previous study, which dealt with the identification of disturbance in BE in VAD patients [1]. Further, we aim to raise awareness of adaptation and integration as two distinct processes in the postoperative course after VAD implantation. Such a conceptual distinction is particularly helpful in psychotherapeutic settings, where both processes should be identified and supported as two sides of the same coin.

Looking at the overall group, the effect of the intervention did not reach statistical significance (*p* = 0.12). Here, it should be noted that inappropriate subjects, that is, patients who are destined to fail, have a negative impact on statistical power [36, 37]. In our case, those are the patients without initial disturbance in BE. Similarly, the tendencies for female (*p* = 0.4) and emergently implanted patients (*p* = 0.24) to demonstrate a less pronounced or no decrease in levels of disturbance in BE from baseline to follow‐up—regardless of group—did not reach statistical significance. Thereby, our results from a previous study that identified women and emergently implanted patients as risk groups could only be confirmed to a limited extent [1]. Again, we identify the low statistical power resulting from the small number of female and emergently implanted patients as an underlying cause.^2^ Reasons for greater disturbance in bodily experience among women may be due to the increased pressure they face to conform to the body ideal [38, 39]. Additionally, compared to men, women‘s self‐concept is more closely tied to perceived physical attractiveness than to physical effectiveness [40, 41]. Accordingly, women are more likely to tend to denial as a coping mechanism, which might reduce their well‐being and Quality of Life. Regarding emergent implantation, the lack of prior emotional and conceptual confrontation seems to initially hinder integrational processes and subsequently cause more profound body image disturbances [42].

Patients are very grateful for the treatment, and the gratitude scores increase slightly over the course of 1 year, albeit not significantly. These overall high gratitude scores are in line with our previous findings [1]. Moreover, the assumptions by Dew et al. about declining patient satisfaction over time, based on decreased patient satisfaction 1 month post‐discharge, can be dismissed [43]. However, no interventional effect on gratitude was observed. This might be explained through the focus of the intervention, which was not aimed at gratitude but BE. Besides, the intervention group exhibited a ceiling effect for “gratitude” in the baseline phase (81% selecting the two highest categories), leaving little room for further improvement at the 1‐year follow‐up (87% selecting the two highest categories).

The independence of the psychological construct BE from demographic variables (such as age) and somatic variables (such as driveline infections) was again confirmed in this study [1]. At first glance, this may contradict clinical experience. However, intuitive expectations can sometimes be deceptive [44].

Last but not least, disturbance in BE is strongly associated with symptoms of anxiety and depression [1, 45]. Consequently, it is reasonable to assume that a focused intervention targeting BE exerts an overall positive impact on mental health.

The findings of this study must be interpreted in the context of some limitations. Firstly, the small number of women and emergently implanted patients limits the power of the corresponding subanalyses. Secondly, standardization is affected by the multicenter nature of the study, since the intervention was necessarily carried out by different psychologists. However, to minimize this effect, all professionals involved in the study attended a one‐day training workshop beforehand. Furthermore, the content was manualized and presented in a curricular way. Thirdly, patients who were not in a stable postoperative condition were excluded to avoid additional strain during their recovery phase. As a result, the findings may not be directly applicable to this subgroup. The same applies to patients with cognitive or linguistic impairments. Fourthly, the relatively high dropout rate in the CG reduced the statistical power of our analyses and limited the ability to detect small effects. The higher dropout rate in the control group likely stemmed from lower involvement and weaker study engagement, as these patients did not receive a specific intervention or additional attention.

## 5 Conclusion

This study highlights the efficacy of targeted psychological support in mitigating disturbance in BE among patients following VAD implantation. In particular, patients who initially exhibited signs of BE disturbance benefit significantly from the intervention, experiencing a marked reduction in BE disturbance 1 year post‐implantation compared to the control group. This finding underscores the importance of tailored therapeutic support for this patient cohort, addressing both integration and adaptation processes associated with VAD implantation. The theoretical distinction between these two processes, which is presented in this work, provides valuable insights for therapeutic practice. In general, patients express profound gratitude immediately after VAD implantation, and this positive attitude persists even after a year of living with the device. In conclusion, our findings emphasize the importance of screening for BE disturbance and providing psychotherapeutic interventions to ultimately improve mental health outcomes in patients with VAD.

## Author Contributions

F.R. contributed to the data analysis, data collection, interpretation and visualization of the data, manuscript draft, and the manuscript writing. W.A. was involved in data collection, study design, review of the data analysis, and writing of the manuscript. H.S., S.S.‐K., K.T.‐L., and C.K. were involved in study design, data collection, and critical revision of the manuscript. C.K. and J.M. interpreted the data and revised the article critically for important intellectual content. All authors approved the final version to be published.

## Conflicts of Interest

The authors declare no conflicts of interest.

## Supporting information


**Data S1.** Supporting Information.

## Endnotes

^1^
We use the term bodily experienced as an inclusive umbrella term that encompasses various aspects of the individual's experience of their body, also including the construct body image. The use of the term “body image” is avoided throughout this work due its conceptual heterogeneity both within and across disciplines.

^2^
The low numbers of VAD implantations in women may result from remaining underdiagnosis of cardiovascular diseases in women [46, 47]. The low incidence of emergency implantations can be attributed to the centers' proactive approach, prioritizing implantation procedures prior to the onset of cardiogenic shock.
