# Supplementary material for: Addressing Disturbance in Bodily Experience After Ventricular Assist Device Implantation: A Multicenter Randomized Controlled Trial of Curricular Psychological Support
Source: Artif Organs. 2025 Mar 17;49(7):1099–107. doi: 10.1111/aor.14996 (PMC12179760; doi:10.1111/aor.14996)
Supplement: Supplementary file 1 — Data S1. Supporting Information. [file AOR-49-1099-s001.docx]

**Addressing disturbance in bodily experience after ventricular assist device implantation: a multicenter randomized controlled trial of curricular psychological support**

Wolfgang Albert^1,2,3^, Hannah Spielmann^4^, Sandra Semmig-Koenze^5^, Christoph Knosalla^1,2,3^, Johanna Mulzer^1,2,3^, Katharina Tigges-Limmer^6^, Christiane Kugler^4^, Fabian Richter^1,2^

^1^ Deutsches Herzzentrum der Charité, Department of Cardiothoracic and Vascular Surgery, 13353 Berlin, Germany;

^2^ Charité – Universitätsmedizin Berlin, corporate member of Freie Universität Berlin and Humboldt-Universität zu Berlin, Charitéplatz 1, 10117 Berlin, Germany;

^3^ DZHK (German Centre for Cardiovascular Research), partner site Berlin, Germany;

^4^ University of Freiburg, Faculty of Medicine, Institute of Nursing Science; Germany

^5^ Leipzig Heart Center; Germany;

^6^ Heart and Diabetes Center North-Rhine Westphalia, University Hospital of the Ruhr University Bochum, Bad Oeynhausen, Germany

**Address of correspondence and reprint requests:**Fabian Richter, PhD
Medical Heart Center of Charité and German Heart Institute Berlin
Augustenburger Pl. 1, 13353 Berlin, Germany
E-Mail: fabian.richter@dhzc-charite.de

Supplementary Data

Supplementary Table 1

| **Characteristics** | **Mean (SD) or n (%)** | | **p-value** |
| --- | --- | --- | --- |
|  | **Intervention group** | **Control group** |  |
| **Gender** |  |  | .89 |
| Male | 36 (83.72 %) | 39 (84.78 %) |  |
| Female | 7 (16.28 %) | 7 (15.22 %) |  |
| **Age** | 55.86 (12.11) | 57.48 (11.59) | .52 |
| **Patient acuity** |  |  | .26 |
| Elective | 39 (90.7 %) | 38 (82.61 %) |  |
| Emergent | 4 (9.3 %) | 8 (17.39 %) |  |
| **Marital status** |  |  | .75 |
| Single | 13 (30.23 %) | 15 (33.33 %) |  |
| Married or partnered | 30 (69.77 %) | 30 (66.67 %) |  |
| **Living situation** |  |  | .78 |
| Alone | 11 (25.58 %) | 13 (28.26 %) |  |
| Not alone | 32 (74.42 %) | 33 (71.74 %) |  |
| **Education (highest)** |  |  | .75 |
| Secondary school | 13 (30.95 %) | 15 (33.33 %) |  |
| High school | 4 (9.52 %) | 2 (4.44 %) |  |
| Vocational education | 17 (40.48 %) | 21 (46.67 %) |  |
| University degree | 8 (19.05 %) | 7 (15.56 %) |  |
| **Kids** |  |  | .84 |
| Yes | 13 (30.23 %) | 13 (28.26 %) |  |
| No | 30 (69.77 %) | 33 (71.74 %) |  |
|  |  |  |  |

Supplementary Table 1. Demographics for the subgroup of patients with baseline BE-S values of ≥ 2. Discrepancies to total are due to missing values; M = Mean, SD = Standard Deviation, n = number of patients.

CONSORT study flow diagram


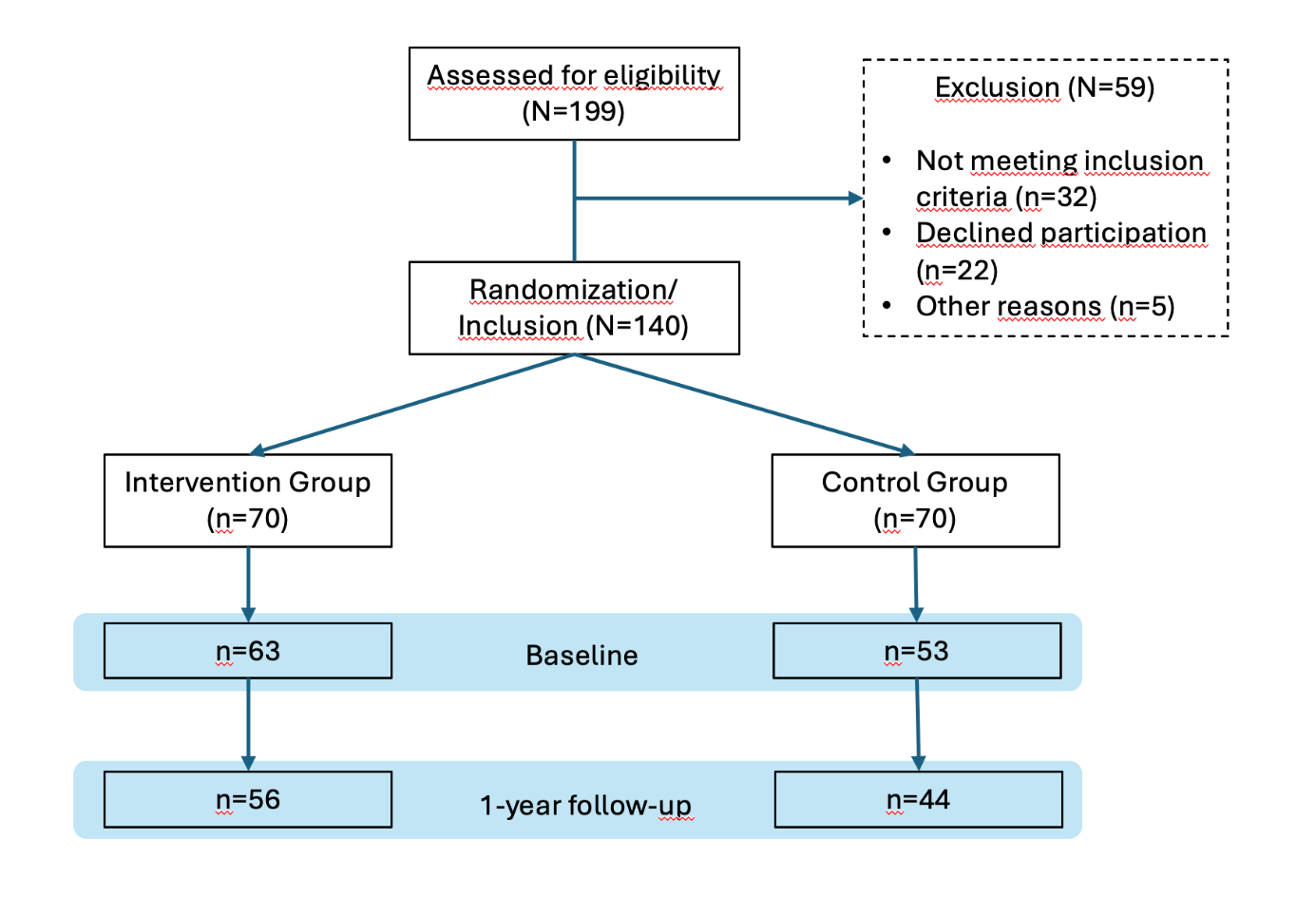


Supplementary Figure 1. CONSORT study flow diagram.

CONSORT study checklist


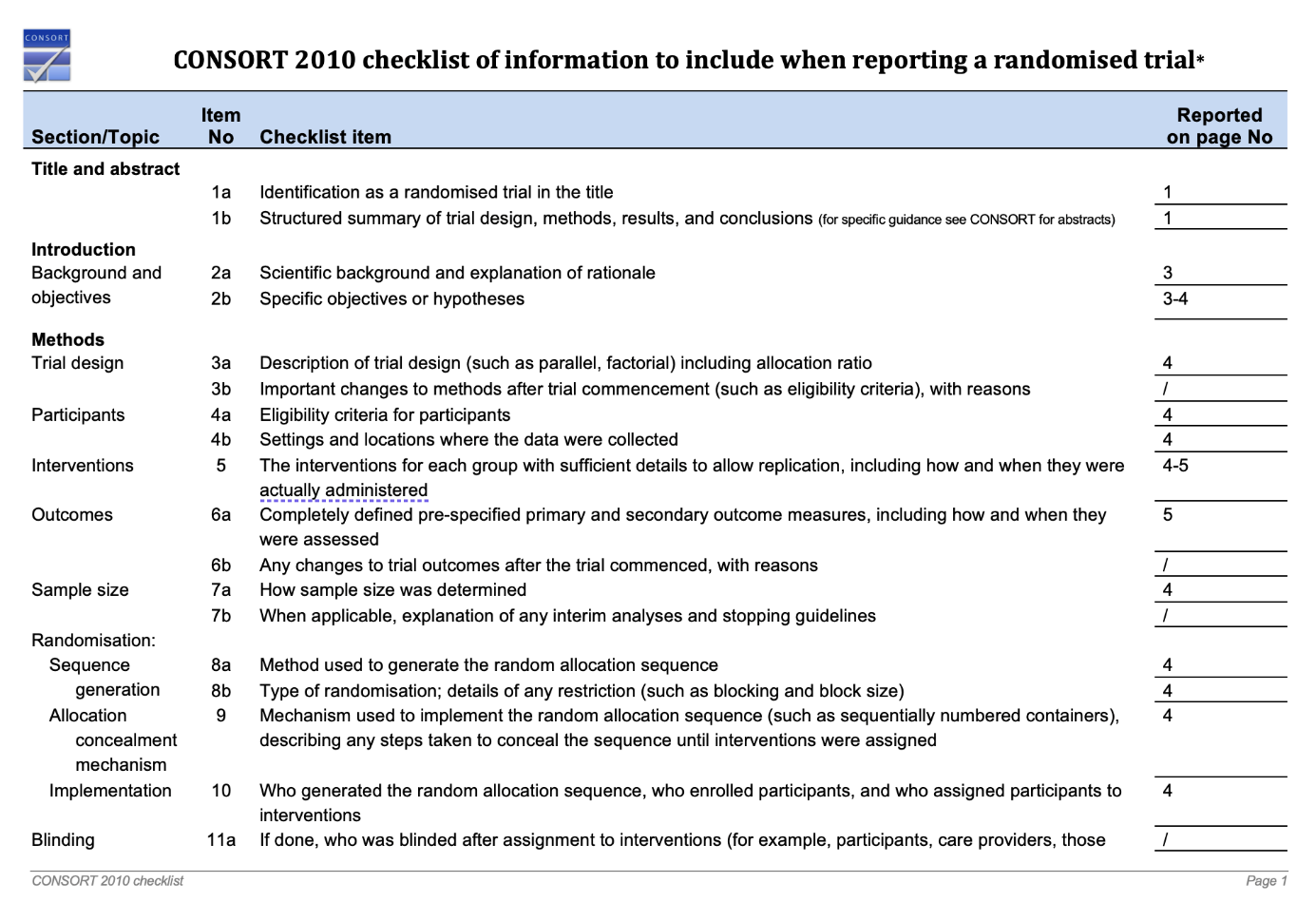


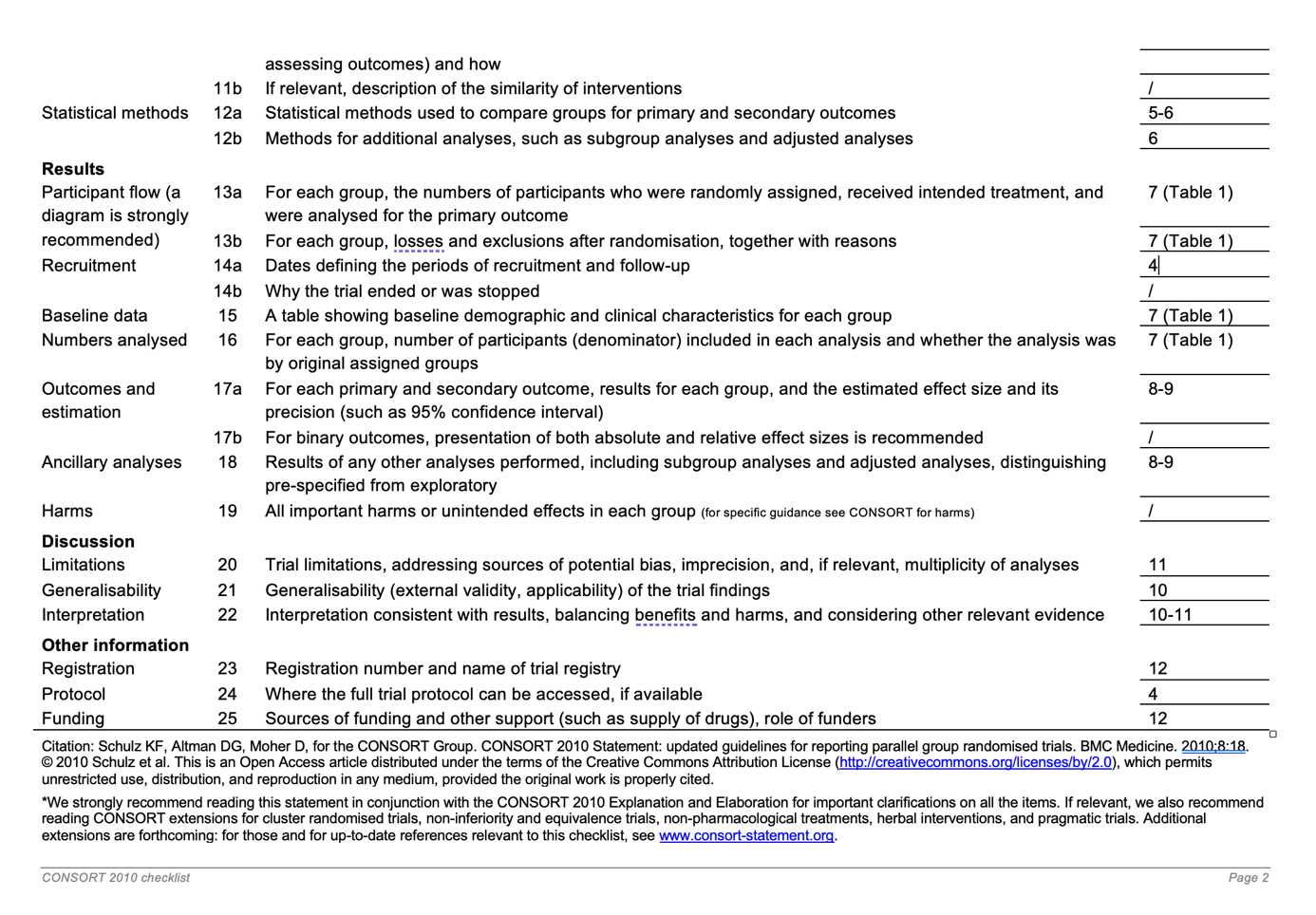


Supplementary Figure 2. CONSORT study checklist.
